# Synthetic Curcuminoid Induces Cell Death in *Trypanosoma cruzi* Through Oxidative Stress and Exhibits Synergistic Activity with Benznidazole

**DOI:** 10.1007/s11686-026-01443-6

**Published:** 2026-11-13

**Authors:** John Washington Cavalcante, Beatriz Maria Silva Martins, Emanuel Paula Magalhães, Naiara Dutra Barroso Gomes, Lyanna Rodrigues Ribeiro, Felipe Ramon Cunha da Silva, Mateus Edson da Silva, Marcelo Morais Gomes Maia, Arif Ali, Márcia Machado Marinho, Emmanuel Silva Marinho, Hélcio Silva dos Santos, Alice Maria Costa Martins, Ramon Róseo Paula Pessoa Bezerra de Menezes

**Affiliations:** 1 Post-Graduate Program in Pharmaceutical Sciences, Federal University of Ceará, Fortaleza, CE Brazil; 2 Post-Graduate Program in Pharmacology, Federal University of Ceará, Fortaleza, CE Brazil; 3 Chemistry Department, Vale do Acaraú State University—UVA, Sobral, CE Brazil; 4 Bioprospecting and Natural Resource Monitoring Laboratory, State University of Ceará, Fortaleza, Ceará Brazil; 5 Department of Clinical and Toxicological Analysis, Federal University of Ceará, Fortaleza, CE Brazil

**Keywords:** Chagas disease, Curcumin, Cell death, Oxidative stress

## Abstract

**Background:**

Benznidazole is currently one of the few drugs available for the treatment of Chagas disease; however, it is associated with significant limitations, including adverse drug reactions and reduced or absent efficacy during the chronic phase. Given the inadequacy of existing therapies, this study investigates the potential therapeutic effect of the synthetic curcuminoid derivative (1E,4E)− 1,5-bis(4-methoxyphenyl)penta-1,4-dien-3-one (DBOCH3) against T. cruzi Y strain using both in vitro and in silico approaches.

**Results:**

In vitro cytotoxicity assays demonstrated that treatment of LLC-MK2 cells with DBOCH3 resulted in a selectivity index of 36, which is 15 times higher than that of benznidazole. Furthermore, DBOCH3 exhibited inhibitory activity against the main life forms of T. cruzi: epimastigotes, trypomastigotes, and amastigotes. Checkerboard matrix assays based on the Bliss independence model revealed that the combination of DBOCH3 and benznidazole exerted a potent synergistic effect against both epimastigote and trypomastigotes forms, particularly within specific concentration combinations, without induced significant toxicity in LLC-MK2 host cells. Flow cytometry analysis indicated that DBOCH3 was associated with parasite cell-death events indicated by 7-AAD and Annexin V-PE labeling, accompanied by cytoplasmic ROS accumulation and loss of mitochondrial transmembrane potential. In silico molecular docking studies revealed that DBOCH3 was predicted to interact with selected T. cruzi enzymes, with predicted binding poses in TcGAPDH and trypanothione reductase (TcTR), with more favorable (more negative) ΔG values than benznidazole.

**Conclusion​:**

These findings indicate that DBOCH3 appears to possess promising characteristics, suggesting that its synergistic potential with benznidazole might offer a viable strategy to enhance antichagasic efficacy and mitigate drug-associated toxicity.

**Supplementary Information:**

The online version contains supplementary material available at https://doi.org/10.1007/s11686-026-01443-6.

## Introduction

Chagas disease (CD), caused by *Trypanosoma cruzi*, is a neglected tropical disease that affects over 6 million individuals, primarily in Latin America but also spreading to other continents, including Europe and North America [1]. CD is associated with high morbidity and mortality, attributable to factors such as strain virulence and the limited efficacy of benznidazole (Bz) during the chronic phase. Moreover, Bz is linked to severe adverse reactions, which restrict its long-term use [2–4]. These adverse effects include cutaneous reactions (such as dermatitis and rash), gastrointestinal intolerance, and peripheral neuropathy; hematological and hepatic abnormalities have also been reported [5]. In this scenario, there is a vital need to develop novel antichagasic drugs, with a primary emphasis on natural compounds and on disrupting key proteins essential for parasite homeostasis.

Natural products are relevant starting points for antiparasitic drug discovery because they provide structurally diverse scaffolds and biologically active motifs that can be modified to improve potency and selectivity [6, 7]. *Curcuma longa*, commonly known as “turmeric”, is a shrub native to India, South Asia, and Indonesia. Curcumin [(1*E*,6*E*)− 1,7-bis(4-hydroxy-3-methoxyphenyl)hepta-1,6-diene − 3,5-dione] is the main phytocompound of *C. longa* extract [8], and presents several biological properties, such as anticancer [9], anti-inflammatory and antioxidant [10], antimicrobial [11, 12], and antiparasitic [13, 14].

Despite its multiple bioactivities, curcumin has a poor pharmacokinetic profile, including low solubility, which limits its clinical use. These limitations are associated with the presence of a diketone moiety. Therefore, synthetic curcuminoids containing a monocarbonyl group have been synthesized to obtain molecules with similar bioactivities and enhanced physicochemical properties [15, 16].

Evidences in the literature demonstrates that curcumin exhibits a broad spectrum of biological properties, highlighted by its proven antiparasitic activity [17]. Furthermore, various synthetic analogues and curcuminoids have emerged as promising trypanocidal agents. This has prompted strategies based on molecular modifications to optimize their pharmacological properties [18]. Within this context, the development of novel monocarbonyl derivatives aims not only to enhance trypanocidal potency compared to the parent compound but also to direct biological activity toward essential macromolecular targets and structures of *Trypanosoma cruzi*, thereby mitigating the common pharmacokinetic limitations of natural curcumin.

Previous preclinical reviews have examined low-cost candidates and drug-combination strategies for Chagas disease [19, 20]. A study combining benznidazole with inhibitors of *T. cruzi* triosephosphate isomerase reported additive or synergistic effects depending on the inhibitor, while arylidene ketones and thiazolidenehydrazines have also been investigated for activity against *T. cruzi* and *Leishmania spp* [20–22]*.*

Additionally, innovative molecular targets involved in essential metabolic and redox processes in *T. cruzi* represent promising strategies for the discovery of new antichagasic drugs. Glyceraldehyde − 3-phosphate dehydrogenase from *T. cruzi* (TcGAPDH) has attracted considerable attention as a potential therapeutic target due to its essential role in parasite glycolysis, which is particularly relevant to the parasite's energy metabolism. Previous studies have demonstrated the feasibility of identifying TcGAPDH inhibitors through integrated structure-based and pharmacophore-based virtual screening approaches, leading to the identification of promising chemical scaffolds for further optimization and antitrypanosomal drug development [23]. Similarly, *T. cruzi* trypanothione reductase (TcTR) represents an attractive target because of its central role in maintaining the parasite's thiol-redox homeostasis and antioxidant defense system, which is essential for coping with oxidative stress [24].

In this context, this study aimed to investigate the trypanocidal effect of the curcuminoid (1*E*,4*E*)− 1,5-bis(4-methoxyphenyl)penta-1,4-dien-3-one (DBOCH_3_) on *Trypanosoma cruzi* strain Y lifeforms, the cellular events involved and the combined effect with benznidazole.

## Methods

### Synthesis and Characterization of DBOCH_3_

**Scheme 1:**
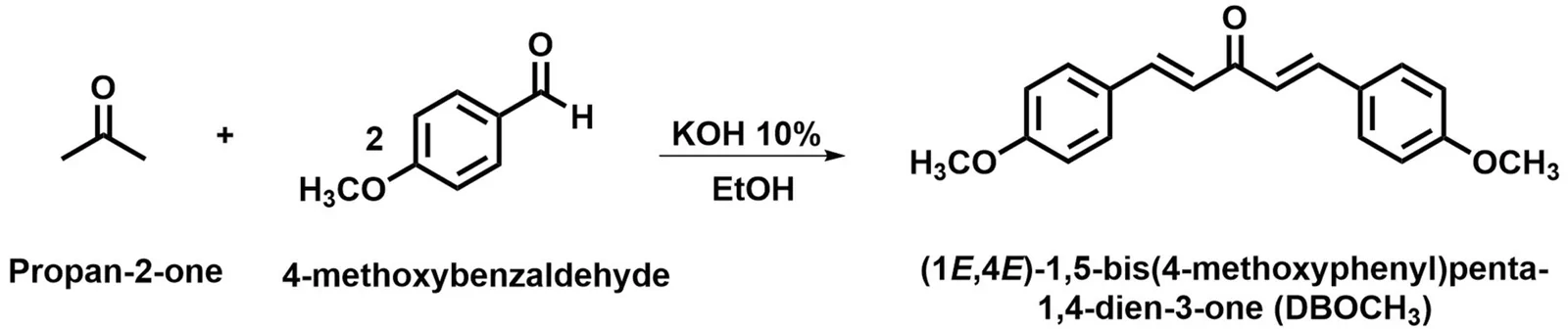
Synthesis reaction of DBOCH_3_

(1*E*,4*E*)− 1,5-bis(4-meoxyphenyl)penta-1,4-dien-3-one (DBOCH_3_) was synthesized by Claisen-Schmidt condensation method[25], with anisaldehyde, as presented in Scheme 1. Briefly, 7 mL of 4-methoxybenzaldehyde:propan-2-one (5:2) was slowly added, with stirring, at room temperature to 50 mL of 10% (w/v) KOH and 40 mL of ethanol. After 30 min, the mixture was filtered, and the precipitate was washed with distilled water. The compound was redissolved in hot ethanol, filtered, and allowed to cool in an ice bath. The crystals formed were washed with cold ethanol and dried at room temperature.

The characterization of the molecule was performed by nuclear magnetic resonance (NMR) spectroscopy. The ^1^H (500 MHz) and ^13^C (125 MHz) NMR spectra were obtained using an Avance DPX-500 spectrometer (Bruker, Billerica, Massachusetts, USA). Spectra were obtained using deuterated chloroform (CDCl_3_) as the reference, and chemical shifts were reported as δ values in parts per million (ppm) relative to CDCl_3_.

Benznidazole (Bz) (C_12_H_12_N_4_O_3_, PM = 260.249 g/mol), kindly donated from Pharmaceutical Laboratory of Pernambuco (LAFEPE), was used as a reference drug. All substances were diluted in dimethyl sulfoxide (DMSO) to prepare stock solutions (0.2 M). For the assays, serial dilutions were prepared in sterile phosphate buffer solution (PBS; NaCl 137 mM, KCl 2.7 mM, KH_2_PO_4_ 1.47 mM, and Na_2_HPO_4_ 8.1 mM, pH 7.4) to obtain working solutions, ensuring that the final DMSO concentration did not exceed 0.5%.

### Cytotoxicity Assay

The cytotoxicity of DBOCH_3_ was assessed in mammalian cells: LLC-MK2 (ATCC CCL-7, monkey kidney epithelial cells). Initially, the cells were plated in sterile 96-well plates (10^5^ cells/mL) in DMEM medium with 10% fetal bovine serum (FBS) and incubated at 37 °C, 5% CO_2_ overnight. After, cells were treated with DBOCH_3_ or Bz (both at 62.5, 125, 250, 500, and 1000 μM) for 24 h. Sterile PBS was used as negative controls, and 0.5% DMSO as a vehicle control. The cell viability was determined using the 3-(4,5-dimethylthiazol-2-yl)− 2,5-diphenyltetrazolium bromide (MTT) assay [26]. Cell viability percentage was calculated relative to the control group, and the data were used to estimate CC_50_ (the concentration that reduces cell viability by 50%) by nonlinear regression.

### Anti-*T. cruzi *in Vitro Activity

The effect of DBOCH_3_ was tested on epimastigote, trypomastigote, and amastigote lifeforms of *T. cruzi* Y strain. This strain was selected for its high infectivity and partial resistance to Bz [27, 28].

Epimastigote forms were cultured in LIT (Liver Infusion Tryptose) medium supplemented with 10% fetal bovine serum (FBS) and antibiotics (penicillin 200 IU/mL and streptomycin 130 mg/L) at 28 °C [29]. For assays, 10^6^ parasites/mL were seeded in 96-well plates and then treated with DBOCH3 or Bz (both at 15.6, 31.25, 62.5, 125, 250, 500, and 1000 μM) for 72 h, and the plates were submitted to the MTT assay. The percentage of viable parasites was used to estimate the concentration that reduces parasite proliferation by 50% (IC_50_).

Trypomastigotes were obtained from the supernatant of infected LLC-MK2 cells, as described [30]. 10^6^ trypomastigotes/mL were cultured in 10% FBS DMEM in 96-well plates and then treated with DBOCH_3_ or Bz (15.6, 31.25, 62.5, 125, 250, 500, and 1000 μM) for 24 h. After that, viable trypomastigotes were counted in Neubauer’s chamber, and the concentration capable to kill 50% of trypomastigote forms (LC_50_) was estimated. Furthermore, the selectivity index (SI) was calculated by the CC_50_/LC_50_ ratio [31].

To obtain amastigote forms, LLC-MK2 cells were cultured on a 24-well plate containing circular coverslips and infected with trypomastigote forms (20 parasites:1 cell) in 2% FBS DMEM. After 48 h, the cells were treated with DBOCH_3_ (62.5, 31.2 and 15.6 µM) or Bz (500 µM). After 24 h, coverslips were washed with sterile PBS, fixed with Bouin’s solution, and stained with Giemsa (5% v/v). The coverslips were examined under light microscopy to determine the percentage of infected cells [32]. Also the antiamastigote activity (AA) was expressed as [33]:

$$\% AA = \left( {1 - \frac{no\;of \;amastigotes/100 \;treated \;cells}{{no\;of \;amastigotes/100 \;untreated \;cells}}} \right) \times 100 $$

### Flow Cytometry Experiments

Flow cytometry experiments were performed with epimastigotes (10^6^ parasites/mL) incubated for 24 h with DBOCH_3_ (125 and 250 µM) in 24-well plates. Data were acquired on a FACS Calibur flow cytometer and Cell Quest Pro software (Becton–Dickinson, California, USA), collecting at least 10^4^ events in each individual analysis.

#### 7-Aminoactinomycin D (7-AAD) and Annexin V-PE Staining

Cell death events induced by DBOCH_3_ were assessed with 7-aminoactinomycin D (7-AAD) and Annexin V-PE [34]. Cells were stained with the BD Pharmingen PE Annexin V apoptosis detection kit I (BD Biosciences) according to the manufacturer's recommendations. Results were expressed as a percentage of non-labeled (viable cells), only-7AAD; only-AxV PE, and double-labeled cells.

#### Cytoplasmic ROS Production and Mitochondrial Transmembrane Potential (ΔΨm)

ROS accumulation was measured with 2′,7′- dichlorofluorescein diacetate (DCFH_2_-DA), which produces green fluorescence proportionally to cytoplasmic ROS accumulation. For that, DCFH-DA (20 µM) was added to the experimental groups after 3 h of incubation with DBOCH_3_. Green fluorescence was measured 24 h later, and the results were expressed as Relative Fluorescence Intensity (RFI), calculated as the ratio between fluorescence intensity in treated and control groups [35].

ΔΨm evaluation was performed using Rho123, which accumulates in viable mitochondria and emits red fluorescence [36]. After 24 h of DBOCH_3_ treatment, the parasites were collected, washed, and stained with Rho123 (10 µg/mL) for 30 min in the dark. Red fluorescence was measured by flow cytometry, and the reduction in mitochondrial potential was assessed using RFI relative to the control group.

### Evaluation of the Synergism Between DBOCH_3_ and Bz in LLC-MK2 Cells

For the cytotoxicity assays, LLC-MK2 cells (10^5^ cells/mL) were cultured in 96-well plates in DMEM supplemented with 10% FBS (37 °C, 5% CO^2^) for 24 h to allow for adherence. Next, cells were treated using an 8 × 8 checkerboard layout, combining decreasing concentrations of DBOCH_3_ on the horizontal axis (20–0.16 μM) and Bz on the vertical axis (10 to 0.08 μM). Separate wells containing the respective individual concentrations of each compound were also included. Cell viability was measured using MTT assay.

### Evaluation of the Synergistic Effect of DBOCH_3_ and Bz in Epimastigote and Trypomastigote Forms

To evaluate the synergistic effect between DBOCH_3_ and benznidazole (Bz), assays were performed using both epimastigote and trypomastigote forms of the parasite. For the epimastigote assay, parasites (10^6^ parasites/mL) were incubated in 96-well plates with combinations of serial concentrations of DBOCH_3_ (range: 20–0.16 μM) and Bz (range: 10–0.08 μM). The experimental design followed an 8 × 8 matrix layout (checkerboard format). Concurrently, separate wells were treated with the same individual concentrations of each compound to serve as control groups for the synergistic concentrations. Cell viability was determined using the MTT assay, and absorbance was measured at 570 nm using a spectrophotometer (Asys Expert Plus®).

Similarly, the synergy assay on trypomastigote forms was conducted in 96-well plates seeded at a density of 10^6^ parasites/mL in DMEM supplemented with 10% fetal bovine serum. Treatment consisted of combinations of serial concentrations of DBOCH_3_ (range: 20–1.25 μM) and Bz (range: 10–0.62 μM) arranged in a 5 × 5 matrix. Both DBOCH_3_ and Bz were also tested individually at the same concentrations. The plates were incubated at 37 °C in a 5% CO_2_ atmosphere for 24 h. Parasite quantification was performed via MTT assay (for epimastigotes) or by counting of viable parasites in Neubauer chamber (for trypomatigotes).

### Molecular Docking

In silico studies were used to evaluate the probable interactions between DBOCH_3_ and *T. cruzi* enzymes: TcGAPDH (glyceraldehyde 3-phosphate dehydrogenase) and Trypanothione Reductase (TcTR). For this, their crystallographic structures were obtained from the RCSB Protein Data Bank (https://www.rcsb.org/). The enzyme TcGAPDH is described as “Structure of *Trypanosoma cruzi* glyceraldehyde − 3-phosphate dehydrogenase complexed with Chalepin, a coumarin derivative inhibitor (BRZ)” (PDB: 1K3T)[37], while TcTR structure is presented as “Crystal structure of *Trypanosoma cruzi* trypanothione reductase in complex with the inhibitor Quinacrine Mustard (QUM)” (PDB 1GXF)[38]. Complex ligands and interferents were removed with AutoDock Tools® version 4.2 (https://autodock.scripps.edu/download-autodock4/) and UCSF Chimera® version 1.13.1 (https://www.cgl.ucsf.edu/chimera/) software. TcGAPDH is a homotetramer and TcTR is a homodimer; the chain identifiers A–D for TcGAPDH and A–B for TcTR refer to their individual subunits.

Docking simulations were performed in AutoDock Vina version 1.2.0 using the Lamarckian genetic algorithm. The grid box was centered to cover all protein chains for TcGAPDH [center coordinates: 19.822 (x), 0.455 (y), and 24.804 (z); size parameters: 116 Å (x), 112 Å (y), and 122 Å (z)] and TcTR [center coordinates: 44.196 (x), 3.102 (y), and − 0.054 (z); size parameters: 126 Å (x), 90 Å (y), and 126 Å (z)]. The docking protocol was first validated by redocking the co-crystallized ligands BRZ and QUM into their corresponding protein structures. Additionally, fifty independent simulations were performed, with twenty poses each [39]. RMSD (root mean square deviation) values were calculated, and values < 2.0 Å were used as the validation criterion [40].The best simulations were selected based on affinity energy (ΔG <  − 6.0 kcal/mol) [41].

### Statistical Analysis

All assays were performed in three independent experiments, with n = 3 in each experiment. Data were expressed as mean ± standard error mean (SEM), and compared by One-way or Two-way ANOVA, with Dunnett’s post-test (*p < 0.05 as significance criteria). Statistical analysis was performed using GraphPad Prism 6.0. Synergy experiments were analyzed using Combenefit software, using the Bliss independence model.

## Results

### Structural Determination of DBOCH_3_

In the ^1^H NMR spectrum, two doublets can be observed at 6.95 (Hα) and 7.69 (Hβ) ppm (J = 15.9 Hz), corresponding to the unsaturated α,β-hydrogens, whose coupling constant (J) confirms the E stereochemistry of the double bond. The signals at 6.92 (d, J = 8.7 Hz, H2/6) and 7.56 (d, J = 8.7 Hz, H3/5) refer to the aromatic hydrogens. In the ^13^C NMR spectrum, the signal referring to the unsaturated α,β-carbonyl at 188.9 was also reported. The ketone absorbs at 203.8; however, the presence of the α,β-unsaturation causes a shift to high field, and the probable cause is the charge delocalization by the benzene ring or the double bond that makes the carbon of the carbonyl less electron-deficient. The α and β olefinic carbons are observed at 127.7 and 142.8. At 161.7 (C-4), 130.2 (C-1), 123.6 (C-3/5), 114.5 (C-2/6) signals confirm the presence of aromatic carbons. The NMR data and the spectra obtained for the molecule are presented in the Supplementary file (Table S1 and Figs. S1–S4).

### Cytotoxicity and Anti-*T. cruzi* Activity

**Fig. 1:**
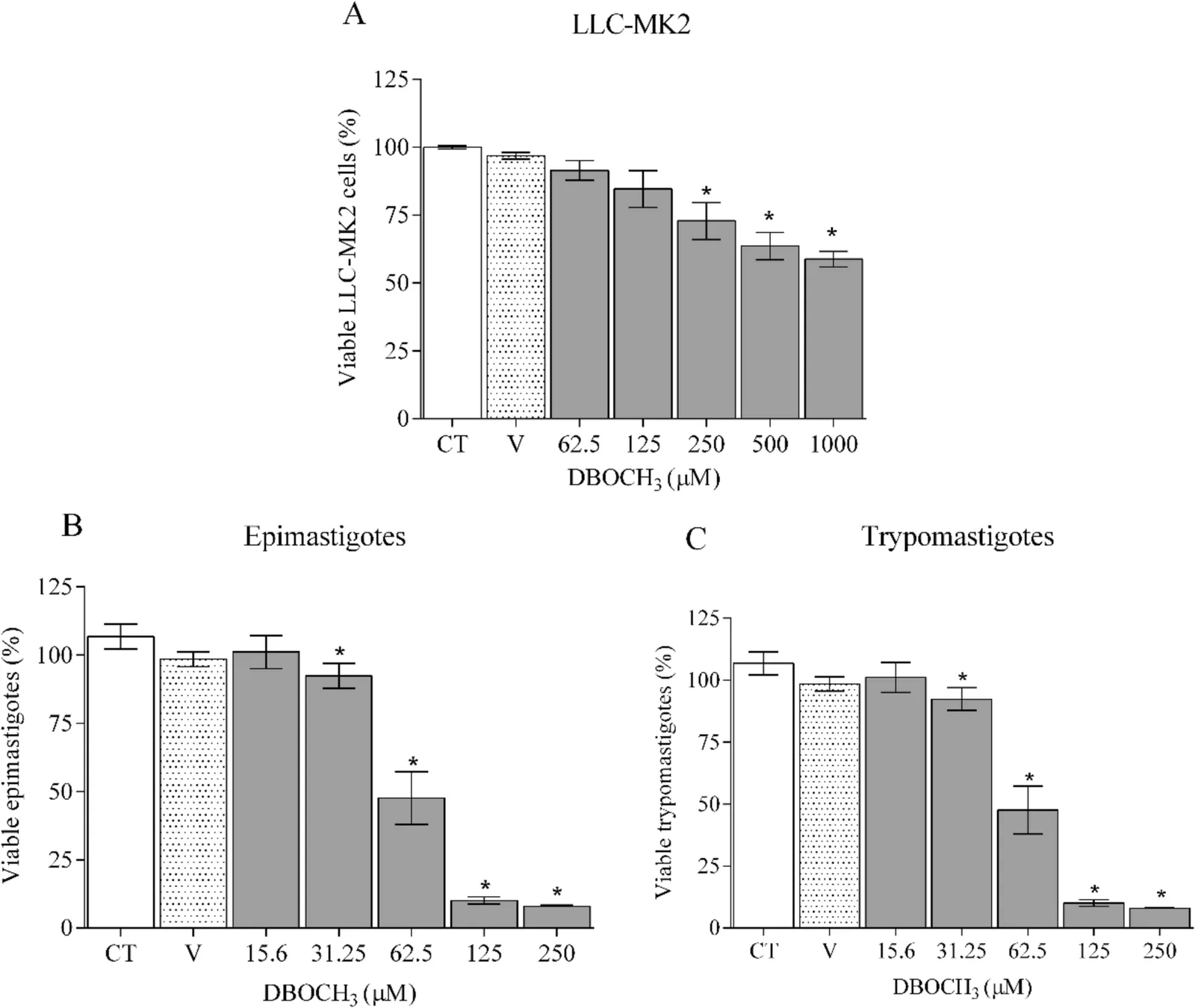
Biological activity of DBOCH_3_ on LLC-MK2 cells, epimastigotes and trypomastigotes of *T. cruzi*. **A** Cytotoxicity on LLC-MK2 cells; **B** Antiproliferative effect on epimastigotes; **C** Antiparasitic effect on trypomastigotes. CT—Control; V—0.5% DMSO. Data are expressed by mean ± SEM. Statistical analysis was performed by One-way ANOVA with Dunnett post-test. *p < 0.05 vs. CT

To assess the DBOCH_3_ cytotoxicity, LLC-MK2 host cells were submitted to MTT assay after treatment for 24 h. DBOCH_3_ reduced cell viability only at the three highest concentrations (Fig. 1A), presenting CC_50_ = 1277 ± 348 µM (Table 1).

**Table 1:** Cytotoxicity, antiproliferative, trypanocidal effect, and selectivity of DBOCH_3_ and Bz

	CC_50_ (24 h) LLC-MK2	IC_50_ (72 h) Epimastigotes	LC_50_ (24 h) Trypomastigotes	Selectivity Index (SI)
DBOCH_3_	1277 ± 348 µM	61.8 ± 0.83 µM	35.5 ± 0.23 µM	36.01
Benznidazole	616 ± 51 µM	16.5 ± 3.0 µM	256 ± 77 µM	2.4

CC_50_ = concentration at 50% cytotoxicity; IC_50_ = growth inhibition at 50%; LC_50_ = inhibitory concentration at 50%; SI = selectivity index. Data are expressed as mean ± SD

In epimastigotes and trypomastigotes, DBOCH_3_ caused antiparasitic activity at most tested concentrations (Fig. 1B), with IC_50_ = 61.8 ± 0.83 µM (epimastigotes) and LC_50_ = 35.5 ± 0.23 µM (trypomastigotes). CC_50_, IC_50_, LC_50_ and SI values obtained for DBOCH_3_ and Bz are presented in Table 1. DBOCH_3_ presented less cytotoxicity and greater trypanocidal effect on trypomastigotes, being 15 times more selective against *T. cruzi* (SI = 36.01) when compared to Bz (SI = 2.40).

**Fig. 2:**
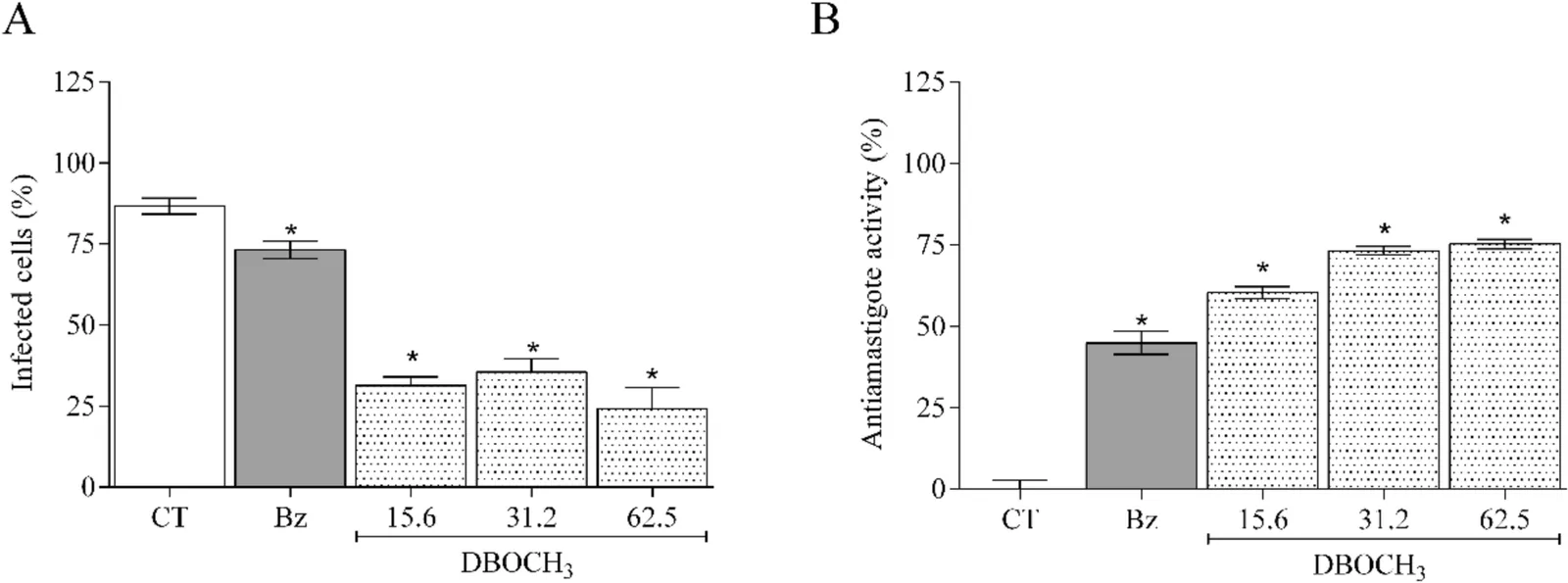
Effect of DBOCH_3_ on infected cells. **A** Effect on infected cells; **B** Antiamastigote activity. CT—Control; Bz—Benznidazole (500 µM). Data are expressed by mean ± SEM. Statistical analysis was performed by One-way ANOVA with Dunnett post-test. *p < 0.05 vs. CT

The analysis on infected cells showed that DBOCH_3_ reduced the percentage of infected cells at all concentrations (Fig. 2A). Considering the number of intracellular amastigotes, DBOCH_3_ presented antiamastigote effect ranging from 60 to 75% (Fig. 2B).

### Assessment of Cell Death Events Induced by DBOCH_3_

**Fig. 3:**
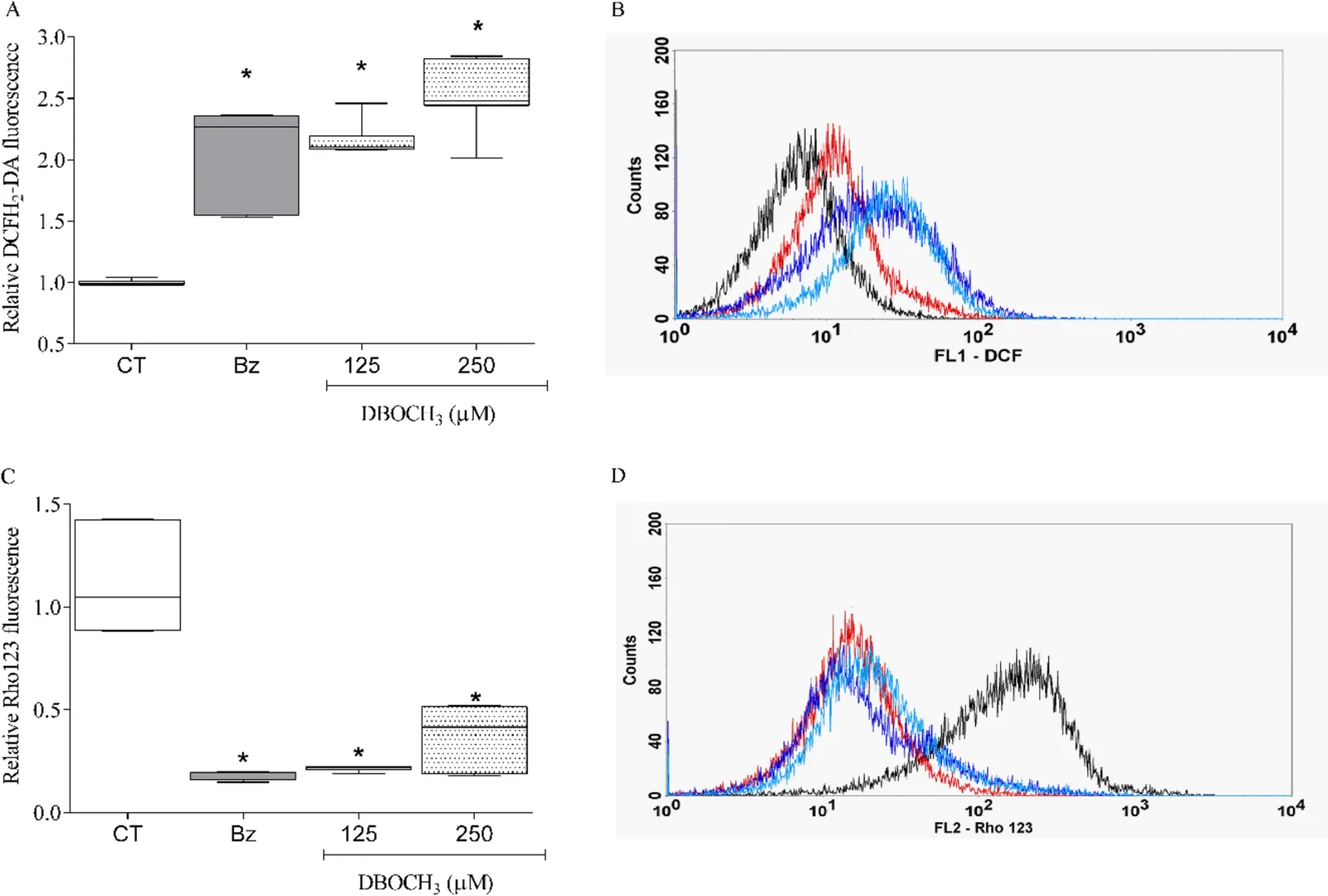
Effect of DBOCH_3_ on reactive oxygen species (ROS) levels and mitochondrial membrane potential (ΔΨm). **A** Relative DCFH_2_-DA fluorescence intensity showing intracellular ROS levels in control cells (CT), benznidazole-treated cells (Bz 500 µM), and cells treated with DBOCH_3_ (125 and 250 µM). **B** Representative histograms for DCFH_2_-DA fluorescence. **C** Relative Rhodamine 123 (Rho123) fluorescence intensity representing mitochondrial membrane potential (ΔΨm) in control cells (CT), benznidazole-treated cells (Bz 500 µM), and cells treated with DBOCH_3_ (125 and 250 µM). **D** Representative histograms for Rho123 fluorescence (FL2—Rho 123). Black—control; red—Bz (500 µM); light blue—DBOCH_3_ 125 µM; dark blue—DBOCH_3_ 250 µM. Data are expressed as mean ± SEM. Statistical analysis was performed by One-way ANOVA (Dunnett post-test) (*p < 0.05 vs. CT)

The cytoplasmic ROS production assay shows that curcuminoid increased DCF fluorescence by more than 200% at the tested concentrations (250 and 125 µM) (Fig. 3A), with a rightward shift of the histogram (Fig. 3B). On mitochondrial transmembrane potential, DBOCH_3_ reduced fluorescence intensity by 72% (Fig. 3C), shifting the histogram to the left (Fig. 3D).

**Fig. 4:**
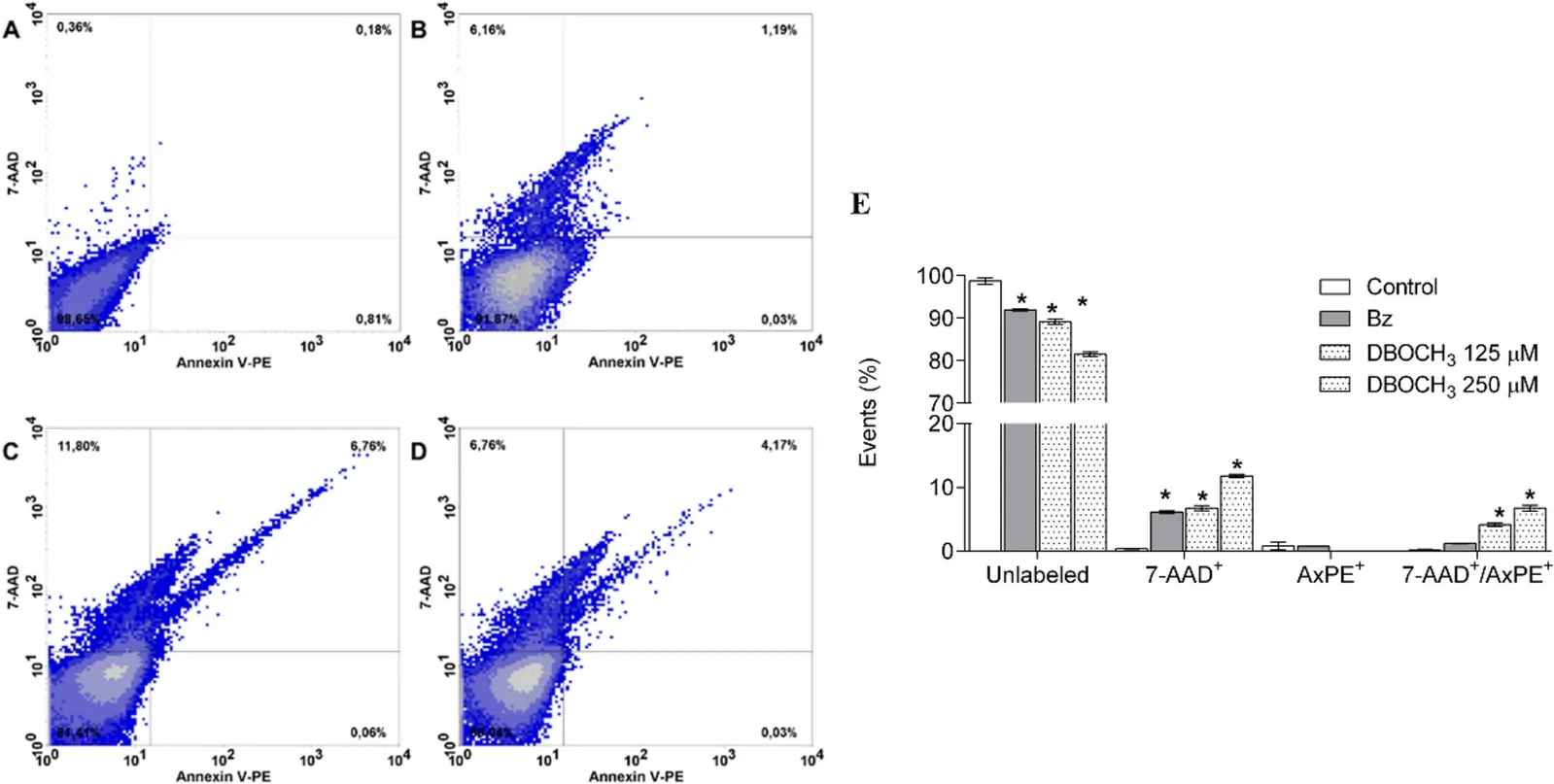
Evaluation of the cell death events induced by DBOCH_3_ on epimastigote forms*.* Dot plot graphs of control **A**, Bz 500 µM **B**, DBOCH_3_ 125 µM (**C**) and DBOCH_3_ 250 µM (**D**). **E** Cell events (%), summarized in 7-AAD^−^/AxPE^−^  = non-labeled/viable cells; 7-AAD^+^/AxPE^−^  = cells without membrane integrity; 7-AAD^−^/AxPE^+^  = phosphatidylserine labeling; 7-AAD^+^/AxPE^+^  = double-labeled cells. Data were expressed as mean ± SEM and analyzed by One-way ANOVA with Dunnet's post-test. (*p < 0.05 vs. CT)

Finally, cells treated with DBOCH_3_ at concentrations 125 and 250 µM have increased only-7-AAD labeling (7-AAD+/AxPE-) on 6.70 and 11.80% of epimastigotes, respectively. At same tested concentrations, DBOCH_3_ increased double-labeling of (7-AAD^+^/AxPE^+^) on approximately 7% of parasites (Fig. 4).

### Synergism Between DBOCH_3_ and Bz in LLC-MK2 Cells

**Fig. 5:**
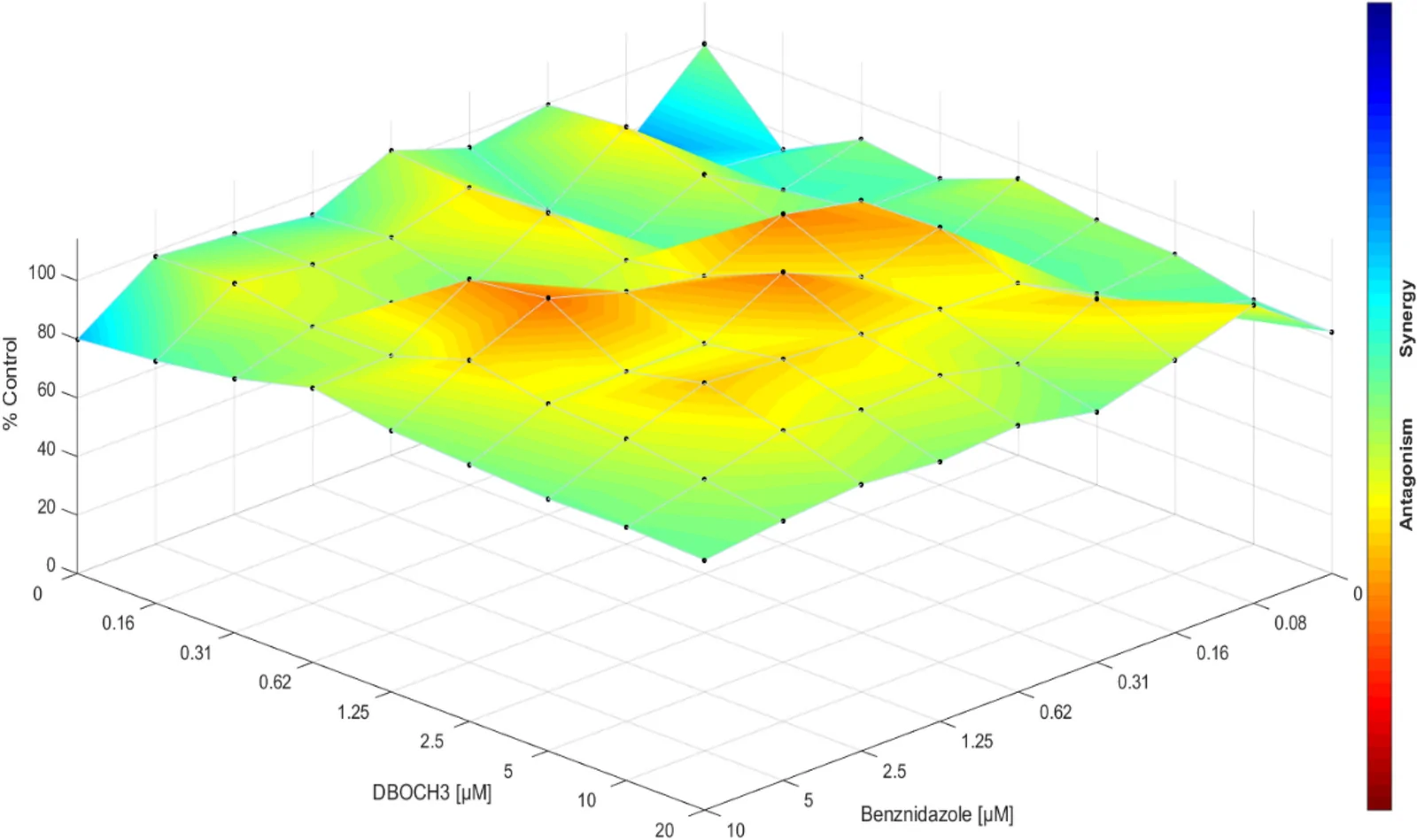
Three-dimensional response surface representing the combined effect of DBOCH_3_ and benznidazole on LLC-MK2 viability. The X- and Y-axes indicate the concentrations of benznidazole and DBOCH_3_ (µM), respectively, while the Z-axis represents parasite viability expressed as percentage of the untreated control. The color scale highlights the interaction profile between the compounds, ranging from antagonism (red/orange) to synergism (blue). Experimental data points from 3 independent experiments are shown as black dots, and the interpolated surface illustrates the overall drug–drug interaction across the concentration matrix

Synergism assays between DBOCH_3_ and Bz in LLC-MK2 cells showed no regions of significant synergism at the tested concentrations, with the exception of a single synergistic point at the 0.16/0.08 μM (DBOCH_3_/Bz) combination (Fig. 5). In the remaining concentrations, regions of antagonism predominated, indicating that these DBOCH_3_ + Bz combinations do not present a considerable cytotoxicity profile against the LLC-MK2 cell line.

### Synergistic Effect of DBOCH_3_ and Bz in Epimastigote and Trypomastigote Forms

**Fig. 6:**
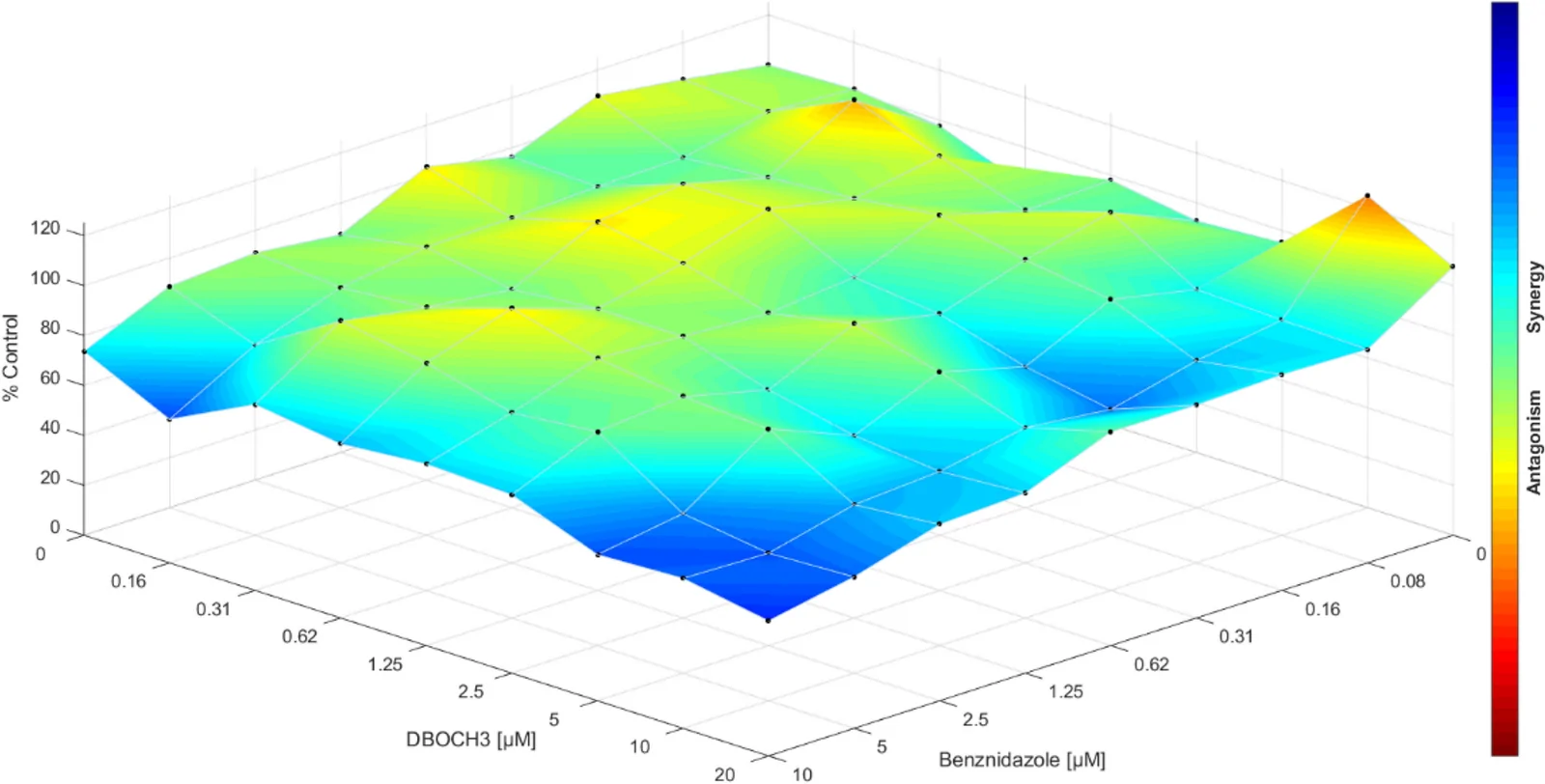
Three-dimensional response surface representing the combined effect of DBOCH_3_ and benznidazole on epimastigotes viability. The X- and Y-axes indicate the concentrations of benznidazole and DBOCH_3_ (µM), respectively, while the Z-axis represents parasite viability expressed as percentage of the untreated control. The color scale highlights the interaction profile between the compounds, ranging from antagonism (red/orange) to synergism (blue). Experimental data points from 3 independent experiments are shown as black dots, and the interpolated surface illustrates the overall drug–drug interaction across the concentration matrix

**Fig. 7:**
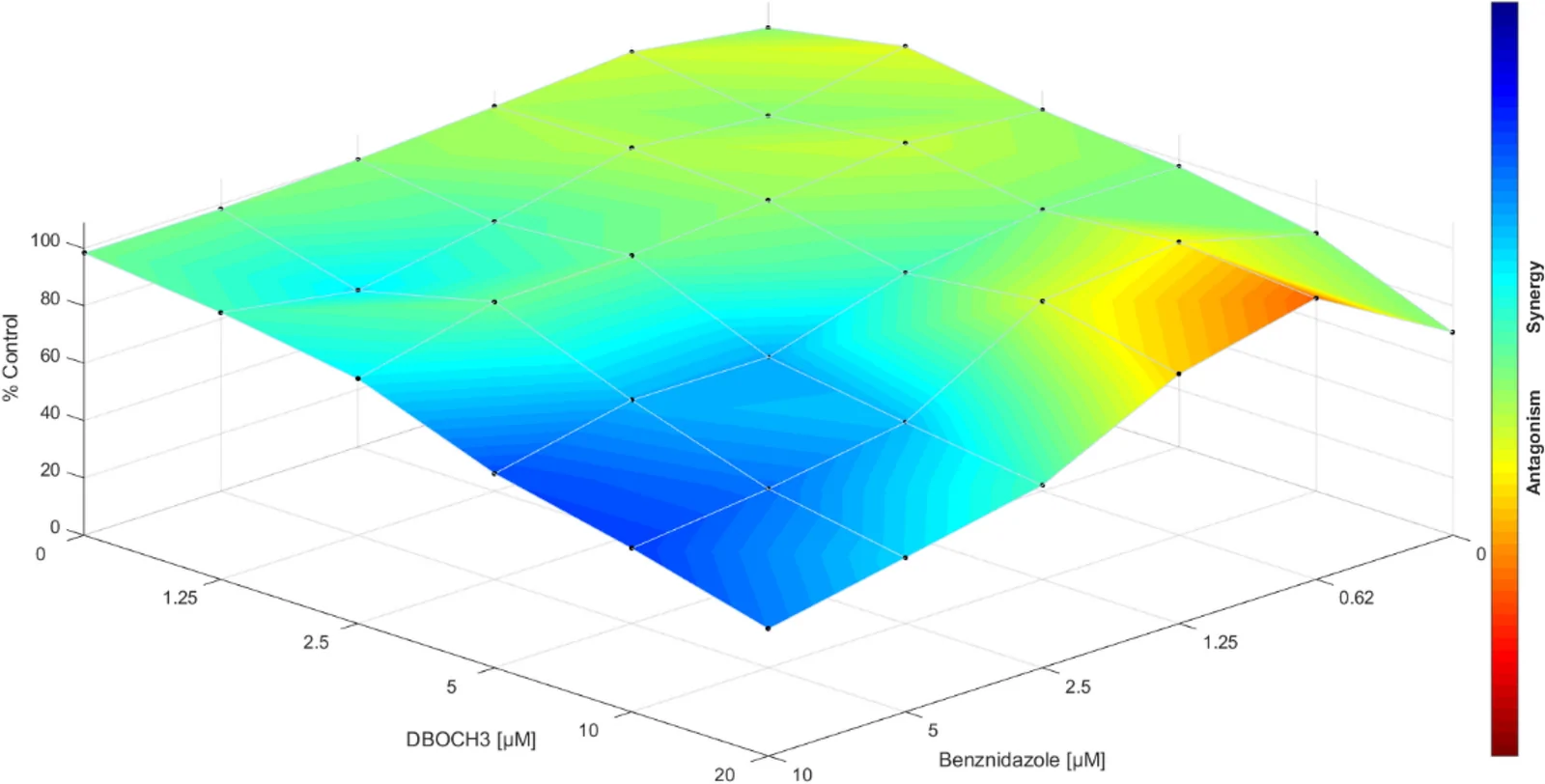
Three-dimensional response surface representing the combined effect of DBOCH_3_ and benznidazole on trypomastigotes viability. The X- and Y-axes indicate the concentrations of benznidazole and DBOCH_3_ (µM), respectively, while the Z-axis represents parasite viability expressed as percentage of the untreated control. The color scale highlights the interaction profile between the compounds, ranging from antagonism (red/orange) to synergism (blue). Experimental data points from 3 independent experiments are shown as black dots, and the interpolated surface illustrates the overall drug–drug interaction across the concentration matrix

Cell growth inhibition was observed in epimastigotes after 72 h of treatment, with evidence of synergism within the concentration ranges of 20–0.5 μM for DBOCH_3_ and 10–0.16 μM for Bz, as highlighted by the deepest dark-blue region on the surface plot (Fig. 6). Based on the synergistic outcomes obtained against the epimastigote forms, the concentrations to be employed in the assays with trypomastigote forms were determined. In the surface plot, the synergistic combinations were concentrated within the ranges of 20–5.0 μM for DBOCH_3_ and 10–5.0 μM for Bz (Fig. 7).

### Molecular Docking

**Fig. 8:**
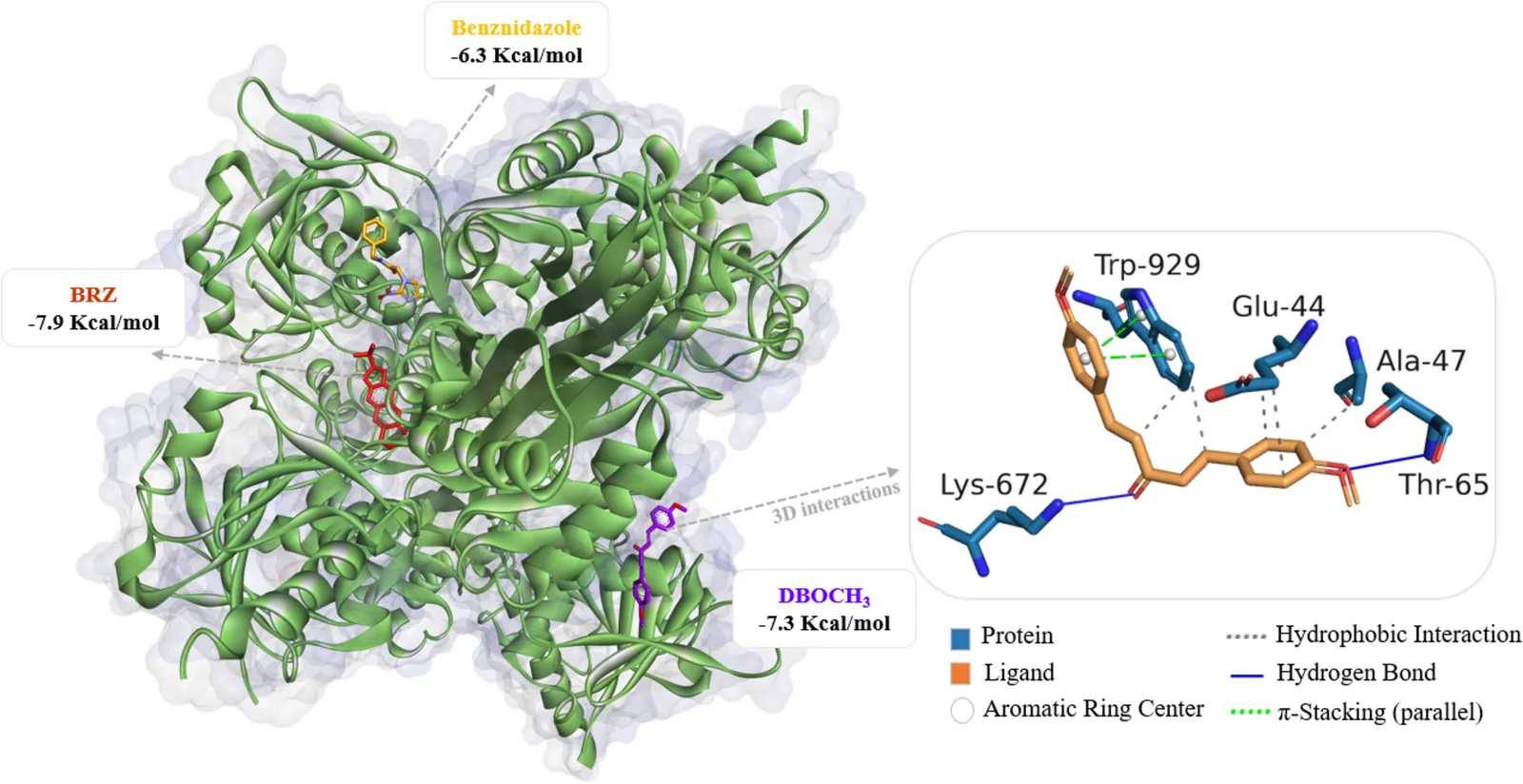
Interaction complex of the enzyme TcGAPDH with the ligands and interactions of the ligand DBOCH_3_ with the receptor

**Fig. 9:**
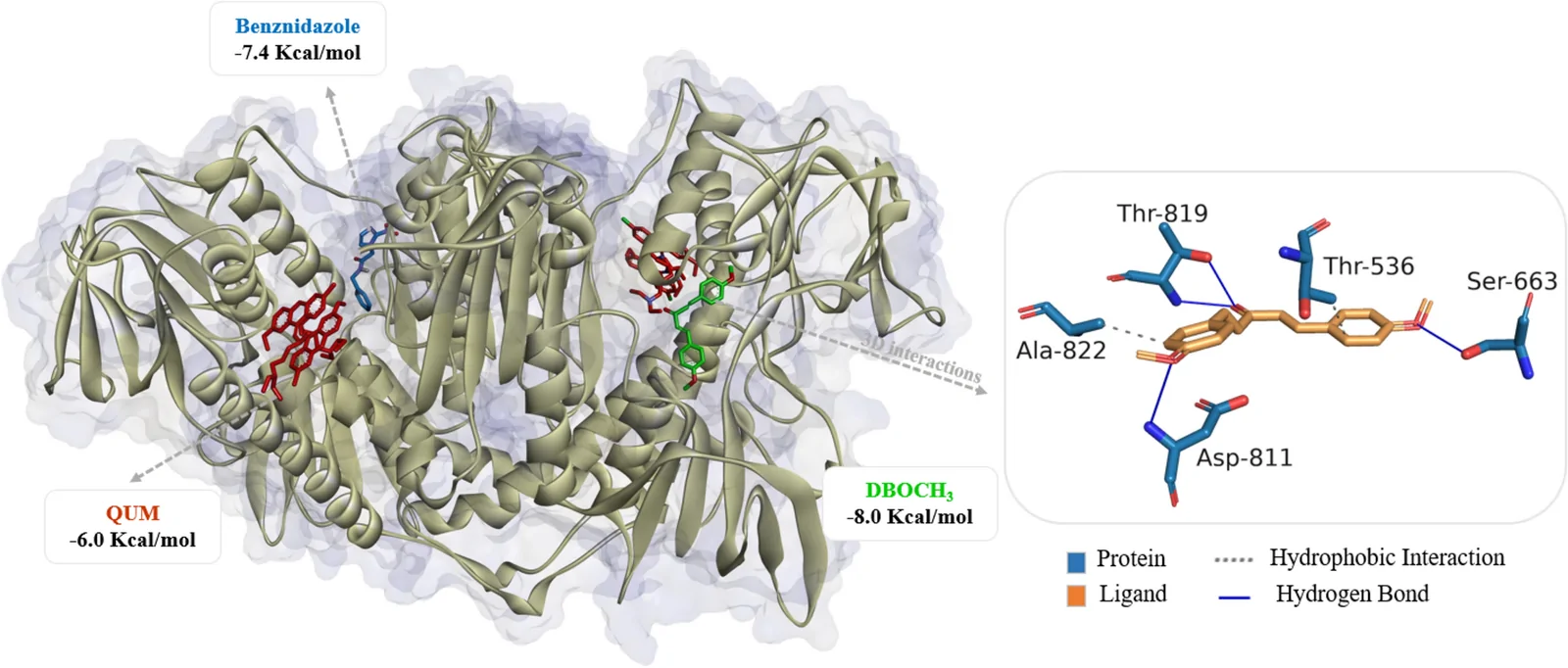
Interaction complex of TcTR enzyme with ligands and interactions of DBOCH_3_ ligand with receptor

The docking analysis aimed to explore possible interactions between DBOCH_3_ and the two selected *T. cruzi* enzymes: TcGAPDH (Fig. 8) and TcTR (Fig. 9). The affinity energy values obtained for the two enzymes (TcGAPDH and TcTR) for the DBOCH_3_ molecule were −7.3 and −8.0 kcal/mol, respectively. It is noteworthy that the curcuminoid showed more favorable ΔG values than Bz (Table 2).

**Table 2:** Affinity energy and RMSD values of the complexes formed by DBOCH3, Bz and co-crystallized inhibitors with the enzymes TcGAPDH, TcTR and TcCr

	TcGAPDH		TcTR	
	ΔG (kcal/mol^−1^)	RMSD (Å)	ΔG (kcal/mol^−1^)	RMSD (Å)
DBOCH_3_	− 7.3	0731	− 8.0	1708
Bz	− 6.3	1758	− 7.4	1352
* BRZ	− 7.9	1920	–	–
* QUM	–	–	− 6.0	1822

Bz, Benznidazole; BRZ, Chalepin inhibitor; QUM, Quinacrine mustard inhibitor; RMSD, Root mean square deviation

The simulations of the DBOCH_3_ -TcGAPDH complex (Fig. 8) showed that DBOCH_3_ formed hydrophobic bonds with the amino acid residues Glu 44 A, Ala 47 A, Trp 929D; hydrogen bonds with Thr 65 A, Lys 672C, and π-stacking with Trp 929D. For Bz, two hydrophobic interactions (Ile 731C, Glu 10 54C), two hydrogen bonds (His 912C and Ser 95C) and one π-stacking (His 912C) occurred (Table 3). DBOCH_3_ interacted in different regions compared to those observed for the inhibitor BRZ.

**Table 3:** Interactions between DBOCH_3_, Bz and BRZ with TcGAPDH

Ligands	AA residue	Interaction	Distânce (Å)	AA residue	Interaction	Distânce (Å)
DBOCH_3_	Glu 44 A	Hydrophobic	3.95	Trp 929D	Hydrophobic	3.87
	Glu 44 A	Hydrophobic	3.69	Trp 929D	Hydrophobic	3.61
	Ala 47 A	Hydrophobic	3.65			
Bz	Ile 731C	Hydrophobic	3.72	Ser 965C	H-bonding	3.17
	Glu 1054C	Hydrophobic	3.64	His 912C	π-stacking	4.13
	His 912C	H-bonding	3.48			
BRZ*	Thr 199C	Hydrophobic	3.90	Thr 167C	H-bonding	2.80
	Asn 335C	Hydrophobic	3.47	Thr 167C	H-bonding	3.05
	Cys 166C	H-bonding	2.79	His 194C	H-bonding	3.95

*Co-crystallized ligand (redocking) BRZ: 6-(1,1-dimethyladyl)− 2-(1-hydroxy-1-methylethyl)− 2,3-dihydro-7 h-furo[3,2-g]chromen-7-one; Bz: benznidazole. The letters next to the amino acid residues indicate the chain to which each residue belongs

The interactions of DBOCH_3_ with TcTR (Fig. 9) revealed hydrophobic interactions with Thr 53 B and Ala 822 B; hydrogen bonds with Ser 3 B, Asp 811 B, and Thr 819 B. Bz showed three interactions: hydrophobic (Ile 200 B), hydrogen-bonding (Thr 335 B), and π-stacking (Phe 199 B) (Table 4).

**Table 4:** Interactions between DBOCH_3_, Bz and QUM with TcTR

Ligands	AA residue	Interaction	Distânce (Å)	AA residue	Interaction	Distânce (Å)
DBOCH_3_	Thr 536 B	Hydrophobic	3.42	Asp 811 B	H-bonding	2.97
	Ala 822 B	Hydrophobic	3.67	Thr 891 B	H-bonding	2.03
	Ser 663 B	H-bonding	2.33	Thr 891 B	H-bonding	2.12
Bz	Ile 200 B	Hydrophobic	3.60	Thr 335 B	H-bonding	2.76
	Thr 335 B	H-bonding	2.95	Phe 199 B	π-stacking	5.00
QUM*	Tyr 111 B	Hydrophobic	3.32	Ser 100 B	Halogen bond	3.15
	Tyr 111 B	H-bonding	3.22	Ser 100 B	Halogen bond	3.61

*Co-crystallized ligand (redocking) QUM: quinacrine mustard; Bz: benznidazole. The letters next to the amino acid residues indicate the chain to which each residue belongs

## Discussion

The present work demonstrates for the first time the trypanocidal effect of the synthetic curcuminoid (1*E*,4*E*)− 1,5-bis-(4-methoxyphenyl)penta-1,4-dien-3-one (DBOCH_3_) on the main lifeforms of *T. cruzi* Y strain, their synergistic effect with Bz and the cell death events involved in this effect. In addition, molecular docking simulations were performed on target proteins essential for *T. cruzi* metabolism and virulence.

Initially, DBOCH_3_ cytotoxicity was estimated on LLC-MK2 host cells, which are commonly used to obtain trypomastigote and amastigote intracellular forms. DBOCH_3_ showed lower cytotoxicity than the reference drug, Bz. After measuring cytotoxicity to host cells, trypanocidal activity experiments were carried out.

On epimastigotes, DBOCH3 presented inhibitory effect on all tested concentrations. Sueth-Santiago et al. [42] evaluated the effect of natural curcuminoids on epimastigote forms over a 7-day treatment period, showing results comparable to those of DBOCH_3_ over 72 h. Furthermore, as these are replicative forms, DBOCH_3_ may interfere with mechanisms involved on parasite survival and replication.

In trypomastigotes, DBOCH_3_ presented antiparasitic effect at lower concentrations than Bz, with an LC_50_ 7 times lower. The estimated LC_50_ value for DBOCH_3_ is very similar to that of the dibenzylideneacetone curcuminoids [43], indicating that this group is more potent than the reference drug. Sueth-Santiago [44] demonstrated that synthetic curcuminoids were able to inhibit the release of trypomastigotes from infected cells. The effect of DBOCH_3_ on trypomastigote forms is of great interest, as these are the infective forms of the parasite found in vertebrate hosts. The antiparasitic effect on these forms is one of the most important strategies for preventing and/or delaying infection of host cells, the consequent differentiation into amastigotes, and the spread of the infection, including the indeterminate/chronic phases [45].

The biological effect of curcuminoids seems to be attributed to the ketone functional group present in the main chain of DBOCH_3_, as the removal of this group compromises the trypanocidal effect [15, 18]. This effect of curcuminoids can be further enhanced by the addition of -OCH_3_ groups in the meta and para positions of the aromatic rings [17, 46]. Furthermore, analogs and monoacetonated hybrids in which these groups were present were more promising against strains of *Leishmania donovani* and *T. brucei* [15, 47, 48].

The trypanocidal activity of DBOCH_3_ may also be associated with the presence of a Michael acceptor group, capable of interacting with macromolecules through the addition of nucleophiles (Michael donors) to activated olefins (Michael acceptors) [49]. In Alkhaldi et al. work [50], the trypanocidal activity of several monocetonic curcuminoids was related to the formation of adducts between trypanothione reductase, an important enzyme for the redox metabolism of the parasite, and monoenone (Michael acceptor). Thus, this bond inactivates the enzyme, decreasing the levels of essential thiols for the parasite's survival, compromising its homeostasis, and leading to cell death [18, 43].

The selectivity of DBOCH_3_ for trypomastigotes was shown to be 15 times greater than that of Bz, with an SI of 36, compared with 2.4 for Bz. This is evident considering its lower toxicity to host cells and the greater increase on its trypanocidal effect. An SI > 10 is considered an excellent criterion for selecting and evaluating a molecule's biological activity [51]. We highlight our high SI value for DBOCH_3_ compared with other studies [51, 52], which may be related to the chemical groups present in its structure. The presence of methoxy groups may be related to this effect, in a similar way to that described by Souza et al. [53] for synthetic derivatives of curcumin: the compounds with the greatest selectivity for *T. cruzi* were those with the highest number methoxy substituents. Thus, the optimum effect of DBOCH_3_ would be related to the presence of two methoxy groups in its molecule.

In the intracellular and replicative forms of *T. cruzi*, DBOCH_3_ reduced the number of infected cells and amastigotes across all concentrations tested. Similar results were observed in the works of Aqeele et al. and Souza et al. [53, 54] *with Leishmania* sp. across similar concentration ranges and treatment times. The antiamastigote effect of DBOCH_3_ is highly relevant, since these parasite lifeforms are the primary ones involved in the spread and progression to the chronic stage of CD [55, 56].

For the flow cytometry experiments, two concentrations of DBOCH_3_ (125 and 250 µM) were selected, corresponding to approximately 2- and 4-fold the estimated IC_50_ value in epimastigotes, respectively. This approach was adopted to facilitate the accurate identification of cellular alterations induced by the compound under investigation. DBOCH_3_ caused damage to the *T. cruzi* membrane after 24 h of treatment, increased cytoplasmic accumulation of ROS, and decreased mitochondrial transmembrane potential. In this way, oxidative stress appears to be one of the key events, since an imbalance between the production and neutralization of ROS can cause peroxidation of membrane lipids, damage to genetic material, and thus affect enzyme systems important for the parasite's survival [57, 58].

Mitochondrial injury can form high-conductance porous channels within mitochondria, leading to a loss of membrane potential and changes in cellular pH. Thus, changes in oxidative phosphorylation and ATP production can lead to the parasite's cell death [59]. DBOCH_3_ was able to decrease the mitochondrial transmembrane potential of *T. cruzi* and induce ROS accumulation in the cytoplasm, as observed in the DCFH_2_-DA and Rho123 assays. These events agree with previous findings, where curcumin was able to inhibit redox balance systems in bovine leukocytes infected by *Theileria annulata* [60]. Similarly, curcumin analogues seem to act in cancer cell lines by production a large amount of ROS, possibly mediated by the Michael acceptor motif [61].

In our results, parasites treated with DBOCH3 showed loss of membrane integrity, a classical feature of necrotic cell death. One of the advantages of necrosis in *T. cruzi* is its irreversible nature, which prevents the parasite from recovering or proliferating and interferes with the maintenance and evolution of the disease [62, 63]. Curcumin and different derivatives have shown effect on the membrane integrity and function in trypanosomatids [52, 64–66].

The synergistic results corroborate other studies in which curcuminoids demonstrated a potent trypanocidal synergistic effect. The combination of curcumin and Bz drastically reduced parasitemia in mice, alongside a reduction in the toxic effects of Bz [6]. Another synthetic molecule derived from curcumin, A3K2A3, exhibited synergistic efficacy when combined with Bz against trypomastigotes, inducing morphological alterations in *T. cruzi* [67].

Other studies have demonstrated a potent synergistic effect of curcumin when combined with genistein [68]; the synthetic rhodesain inhibitor RK-52 [69]; and the dipeptide nitrile CD34 [70] against the rhodesain enzyme of *Trypanosoma brucei* rhodesiense. Recent preclinical reviews summarize low-cost candidates and drug-combination strategies for Chagas disease. The combination of benznidazole with *T. cruzi* triosephosphate isomerase inhibitors produced additive or synergistic effects depending on the inhibitor [71]. Arylidene ketones and thiazolidenehydrazines have also been evaluated for activity against *T. cruzi* and *Leishmania* spp. [22]*.* Furthermore, curcumin combined with chemotherapeutic agents has shown synergism in the treatment of various types of cancers, in addition to enhancing the intracellular concentration of these drugs [72–75].

As is well established in the literature, Bz is a drug with poor tolerability and is considered a considerably toxic molecule [76]. Other studies have also demonstrated, using in *vivo* assays, that the combination of Bz with other substances increased trypanocidal activity while attenuating its toxic effects [77–81]. For instance, the combined administration of benznidazole/itraconazole eliminated bloodstream parasites more efficiently than either drug alone [82].

Finally, we performed molecular docking assays in order to suggest possible molecular mechanisms involved on DBOCH_3_ anti-*T.cruzi* effect. Recently, some targets have been extensively studied, considering their importance for parasite survival and proliferation across its life cycle. Enzymes such as glyceraldehyde − 3-phosphate dehydrogenase (TcGAPDH) and trypanothione reductase (TcTR), have been considered promising targets in preclinical drug-discovery research [83–85]. Our results suggest that the curcuminoid may be interacting with these targets.

TcGAPDH is an enzyme involved in energy production in *T. cruzi*, in addition to its functions in gene expression, maintenance of the parasite's telomere structure, and maintenance of NAD+/NADH levels in the energy and redox balance [83, 84]. Glu44, Ala47, and Trp929 residues with which DBOCH_3_ interacted, although not located in the catalytic site, are in a region close to the Met39 residue, a residue that, in the *Leishmania mexicana* isoenzyme, is involved in the inhibition of the enzyme's activity by compounds present in fractions of the *Malachra alceifolia Jacq*. extract [85].

With TcTR, DBOCH_3_ interacted in a region close to the catalytic site of the enzyme's B subunit, which we hypothesize that could interfere with the coupling of the trypanothione disulfide substrate T[S]_2_ and its conversion into T[SH]_2_, or its coenzymes FADH_2_ and NADH [50, 86]. These interactions occurred through hydrogen bonds between the methoxyl and carbonyl groups of DBOCH_3_.

TcTR is involved in the parasite's survival under oxidative stress throughout its life cycle. In addition, it controls ROS production, since basal levels of these species must be maintained for the parasite's cellular responses, such as differentiation, to function in a coordinated manner. However, inhibition of TcTR in *T. cruzi* remains poorly characterized compared with studies in *Leishmania* sp., where very promising molecules inhibit this target, induce intense oxidative stress, and result in consequent mitochondrial damage. These findings, together with the mitochondrial dysfunction and oxidative stress caused by DBOCH_3_, may reinforce the hypothesis that the molecule interferes with processes crucial to the parasite's homeostasis [38, 50, 83].

Additionally, our in silico data agree with the results from flow cytometry, considering that interference on either TcGAPDH or TcTR activities may help to explain the occurrence of oxidative stress and mitochondrial impairment. Glycolysis inhibition causeb by interference on TcGAPDH activity can cause abrupt decrease on pyruvate accumulation, suppression of flagellar motility and consequent parasite lysis [87]. Also, it can cause ROS overproduction, mitochondrial and plasmic membrane damage [88, 89].

Regarding TcTR, this is an enzyme exclusive to Trypanosomatids, which can be explored as a selective target for new drugs. *T. cruzi*, during its life cycle, deals with many oxidative conditions, and its survival depends on a very efficient antioxidant machinery [57]. The inhibition of trypanothione synthetase, for instance, is lethal for *T. cruzi* survival [90]. This way, TcTR inhibitors can cause lipid peroxidation, oxidative stress, mitochondrial damage, as well triggers different cell death mechanisms [91, 92].

In conclusion, DBOCH_3_ exhibited trypanocidal activity against all life stages of *T. cruzi*, and this effect is associated with the induction of oxidative stress, mitochondrial dysfunction, and damage to the plasma membrane. In addition, this molecule seems to have synergistic effect with Bz, which can be useful in the development of new therapeutic strategies for CD. Thus, these data may explain the trypanocidal effect of DBOCH_3_ and provide prospects for a new anti-*T. cruzi* drug candidate.

## Supplementary Information

Below is the link to the electronic supplementary material.

Supplementary file1 (DOCX 116 KB)

## Data Availability

No datasets were generated or analysed during the current study.
