# Supplementary material for: Synthetic Curcuminoid Induces Cell Death in Trypanosoma cruzi Through Oxidative Stress and Exhibits Synergistic Activity with Benznidazole
Source: Acta Parasitol. 2026 Nov 13;71(6):259. doi: 10.1007/s11686-026-01443-6 (PMC13759718; doi:10.1007/s11686-026-01443-6)
Supplement: Supplementary file 1 [file 11686_2026_1443_MOESM1_ESM.docx]

**Supplementary material**

Figure S1.^1^H NMR (500 MHz, CDCl_3_) spectrum of methoxyphenyl)penta-1,4-dien-3-one (DBOCH_3_).

Figure S2. Expasion of ^1^H NMR (500 MHz, CDCl_3_) spectrum of methoxyphenyl)penta-1,4-dien-3-one (DBOCH_3_).

Figure S1.^13^C NMR (125 MHz, CDCl_3_) spectrum of methoxyphenyl)penta-1,4-dien-3-one (DBOCH_3_).

Figure S1. Expansion of ^13^C NMR (125 MHz, CDCl_3_) spectrum of methoxyphenyl)penta-1,4-dien-3-one (DBOCH_3_).
